# Characteristics of NK cells activation following IL-12, IL-15, IL-18 cytokines stimulation for preclinical purposes

**DOI:** 10.1038/s41598-026-42816-1

**Published:** 2026-03-16

**Authors:** Justyna Czapla, Alina Drzyzga, Ryszard Smolarczyk

**Affiliations:** Center for Translational Research and Molecular Biology of Cancer, Maria Skłodowska-Curie National Research Institute of Oncology, Gliwice Branch, Wybrzeże Armii Krajowej Street 15, Gliwice, 44-102 Poland

**Keywords:** NK cells, IL-12, IL-15, IL-18, Cytokine-induced memory-like NK cells, Cancer immunotherapy, Cancer, Immunology

## Abstract

Adoptive cell therapy is a promising strategy in cancer treatment, among which NK cell therapies represent an encouraging approach. Freshly isolated NK cells exhibit low cytolytic activity. Therefore, efficient activation of NK cells is essential to enhance their anti-cancer properties. The aim of this study was to compare ex vivo NK cells activation with commonly used cytokines, including IL-2, IL-12, IL-15, IL-18, or STING agonist, and to determine the most effective activation method. Our results indicate higher viability and expression of activating receptors and granzyme B in IL-15-treated NK cells. The addition of IL-12 to IL-15 augmented the production of IFN-γ and CCL3 in NK cells. However, incubation of NK cells with a cocktail of IL-12, IL-15, and IL-18 cytokines generated massive production of pro-inflammatory cytokines and a significant increase in activation marker CD69. We have also observed reduced viability of NK cells after incubation with STING agonist. NK cells activated with IL-12/IL-15, and IL-12/IL-15/IL-18 co-cultured with MHC^−^ tumor cells, and in each cytokine combination with MHC^+^ tumor cells caused proliferation inhibition and lysis of tumor cells. Activation of NK cells, particularly with the combination of IL-12/IL-15/IL-18 cytokines, shows potential clinical relevance in adoptive cell therapy.

## Introduction

Natural killer (NK) cells belong to innate lymphoid cells with increasing promise for novel cell-based immunotherapies for cancer. Their principal role is to recognize and eliminate infected and malignant cells that lose the self-distinguishing molecules, like major histocompatibility complex (MHC) class I^1,2^. To execute these functions, NK cells possess several qualities. First, they secrete lytic granules, which contain perforin and granzymes, and produce multiple pro-inflammatory cytokines, e.g., IFN-γ and TNF-α. Then, NK cells induce death receptor-mediated apoptosis, and they are capable of mediating antibody-dependent cellular cytotoxicity (ADCC). Their functions depend on the specific balance of inhibitory and activating receptors, and on the presence of particular cytokines in the environment^3^.

The potent and broad anti-tumor action of NK cells triggered the development of NK cell-based therapies. This approach focuses on boosting NK cells expansion, effectiveness, persistence, and specificity toward tumor cells. One of the strategies to achieve these characteristics is to isolate primary cells and activate them with specific cytokines^4^.

The classical, primary cytokine that supports the development, proliferation, and activation of NK cells is IL-2. However, treatment with IL-2 led to clinical systemic toxicities, may stimulate immunosuppressive regulatory T cells, and the use of IL-2 to pre-activate NK cells before infusion has shown further limitations^5,6^. Another cytokine that shares functional similarities with IL-2 is IL-15. Its presence provides NK cells proliferation and stimulation. However, some tumor-supporting properties of its action were noted. Moreover, chronic stimulation of NK cells with IL-15 led to cells unresponsiveness^7^. While use of IL-2 or IL-15 alone may trigger some adverse effects, the combinatorial pre-activation with IL-12, IL-15, and IL-18 has been revealed as a promising tool for NK cells regulation for adoptive transfer. Such pre-activated cells are known as cytokine-induced memory-like (CIML) NK cells. The studies using IL-12, IL-15, and IL-18 to enhance NK cells functions in vitro demonstrated increased NK cells proliferation and production of IFN-γ^3,8^. Additionally, IL-15 and IL-18 have been recently shown to improve NK cell recovery following cryopreservation^9^.

Regarding the application of NK cells in adoptive cell therapy, the use of CIML NK cells resulted in significant tumor regression observed in pre-clinical models and benefits in clinical trials against acute myeloid leukemia. Additionally, there are several clinical trials currently recruiting patients for CIML NK cells-based therapy (NCT05580601, NCT06321484, NCT06152809, NCT06318871, NCT06138587, NCT03068819, NCT07011004, NCT02782546, NCT05629546, http://www.clinicaltrials.gov)^10,11^.

The encouraging anti-cancer properties of the NK cells place great emphasis on the need to develop protocols for their optimal isolation and expansion ex vivo for cell therapy strategies. Thus, here, we used NK cells isolated from the spleens of two mouse strains to assess their viability, immunophenotype, cytokine repertoire, and cytotoxicity following activation with IL-2, IL-15, IL-12, IL-18, or STING agonist, and their paired and triple combinations.

In the current study, following IL-12, IL-15, and IL-18 treatment, we confirmed the potent production of IFN-γ and other pro-inflammatory and chemoattractant cytokines in NK cells. Such treated cells characterized an activated immunophenotype and the ability to induce cancer cells lysis, which together prove their applicability in adoptive cell therapy.

## Results

### Magnetic separation of NK cells

**Fig. 1:**
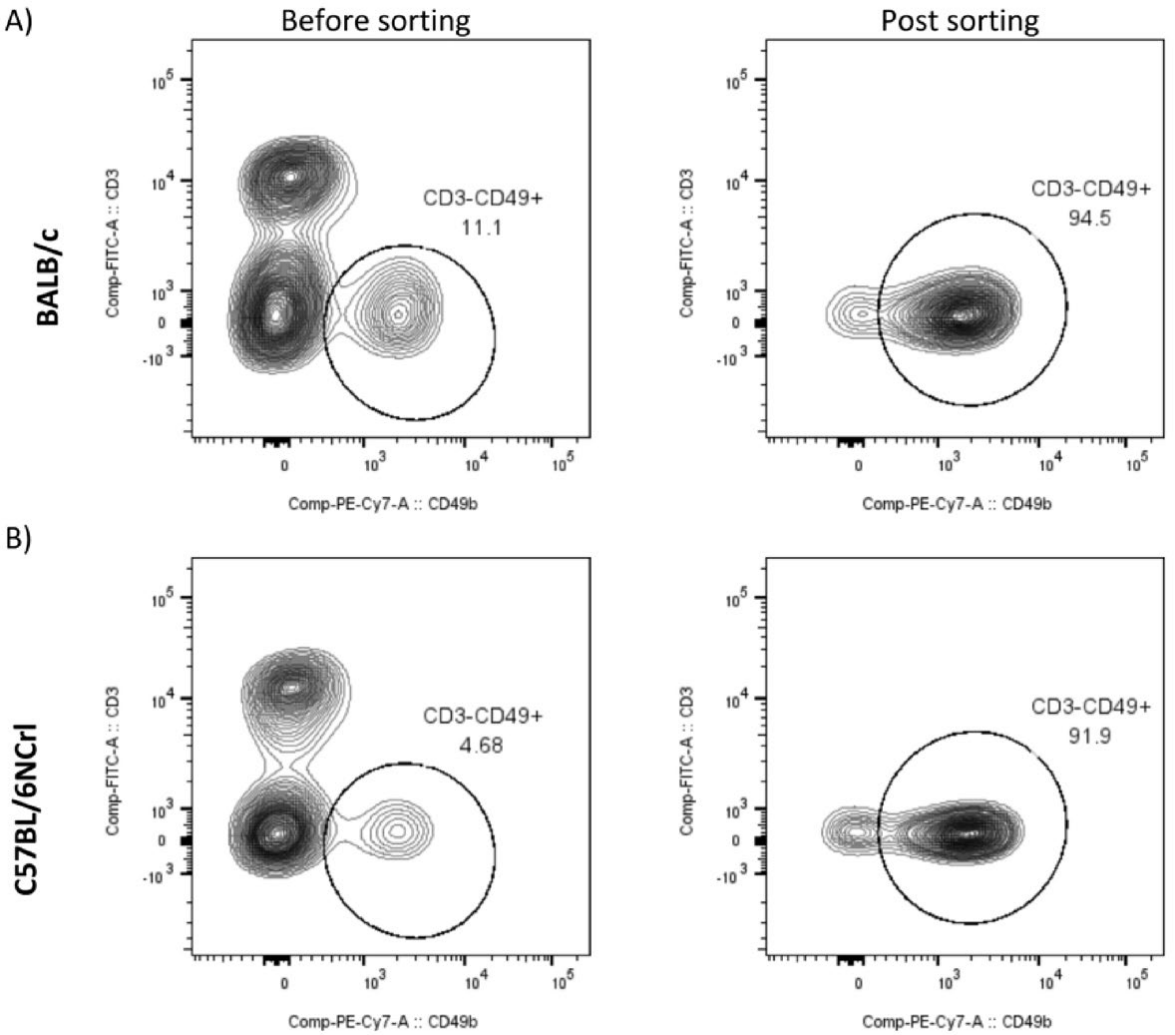
Representative images of contour plots show the purity of NK cells isolated from **(A)** BALB/c mice **(B)** C57BL/6NCrl mice spleens and separated on MACS Magnetic Separator. Contour plots show the percentage share of NK cells (CD3^−^CD49b^+^ cells) in splenocytes (left panel) and the purity of the obtained NK cells population following magnetic separation (right panel).

NK cells were isolated from spleens collected from mice of two strains: BALB/c and C57BL/6NCrl. The total number of splenocytes obtained for magnetic separation differed between the two mouse strains. The number of splenocytes was higher from the spleens of BALB/c mice (9.2 × 10^7^ cells from one spleen) compared to C57BL/6NCrl strain (7.2 × 10^7^ cells). Concomitantly, the percentage of NK cells (gated as CD3^−^CD49b^+^) in splenocytes isolated from the spleens of BALB/c strain was higher than that of C57BL/6NCrl strain (11% vs. 4%) (Fig. 1). The NK Cell Isolation Kit (Miltenyi Biotec) was used to isolate untouched NK cells from a single-cell suspension of splenocytes. The purity of NK cells separation exceeded 90% (Fig. 1). The number of obtained pure NK cell population from BALB/c spleens was higher compared to C57BL/6NCrl spleens (the yield from BALB/c strain was 2.7% and from C57BL/6NCrl 1.6%).

### The phenotype and survival of isolated NK cells

**Fig. 2:**
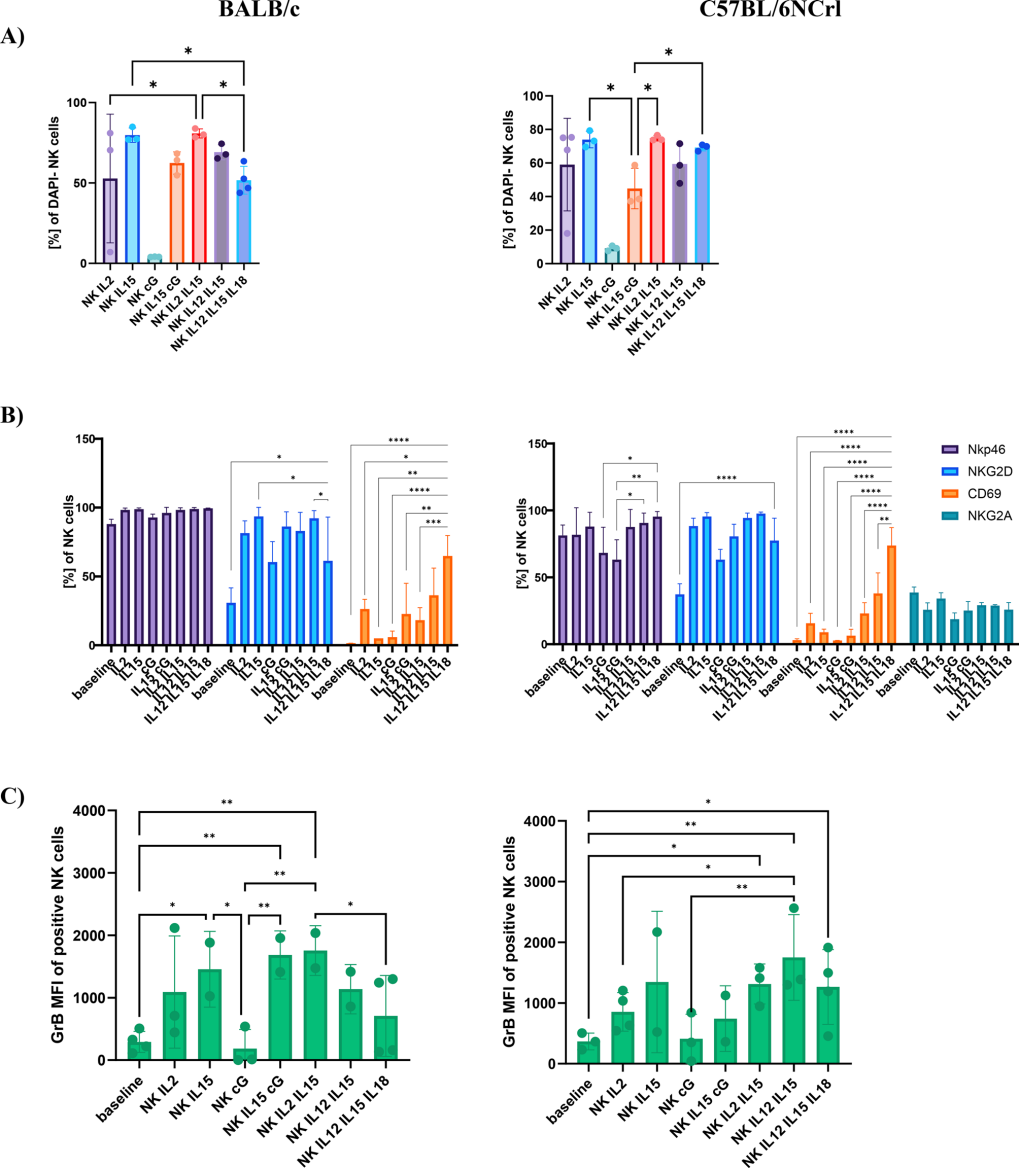
The survival **(A)** and phenotype **(B)**, **(C)** of NK cells isolated from spleens of BALB/c mice and C57BL/6NCrl mice. NK cells were separated on the MACS Magnetic Separator and cultured for 24 h with cytokines indicated on the X-axis, baseline refers to samples of freshly isolated, uncultured NK cells. (A) The percentage of dying cells was assessed using the FACS CANTO II cytometer, gated as DAPI^+^CD3^−^CD49b^+^ cells. (B) The surface receptors were assessed using the FACS CANTO II cytometer; the percentage is shown as a percentage of live CD3^−^CD49b^+^ cells. (C) NK cells were fixed and permeabilized, and the mean fluorescence intensity (MFI) of granzyme B (GrB) was assessed from the population of CD3^−^CD49b^+^ cells. The statistical significance: (A) (C) one-way ANOVA with uncorrected Fisher’s LSD test *p < 0.05;(B) two-way ANOVA with Tukey’s multiple comparison test *p < 0.05; **p < 0.01; ***p < 0.001; **** *p* < 0.000; *n* = 3–4.

Following separation, NK cells were cultured for 24 h in complete medium with the addition of cGAMP or cytokines commonly used for NK cells cultivation: IL-2, IL-12, IL-15, and IL-18. Our aim was to directly compare the effects of cytokines and cGAMP on NK cells survival and receptor expression in two commonly used preclinical mouse strains. We found that 24-hour incubation with cGAMP led to a decrease in NK cells survival (over 90% mortality in both strains). The best survival was exhibited by NK cells incubated with IL-15, both as a single activating cytokine and in combination with IL-2 (almost 80% of survival). NK cells incubated for 24 h with IL-2 showed slightly worse survival (60%), similar to cells incubated with a combination of IL-12/IL-15 and IL-12/IL-15/IL-18 (Fig. 2A).

We checked the phenotype of differentially activated NK cells. We evaluated the expression of activating receptors: NKp46 (CD335), NKG2D, CD69, and inhibitory receptor NKG2A (expressed only in C57BL/6NCrl strain) (Fig. 2B). In BALB/c strain, NKp46 expression was at a similar level in all culture conditions, whereas C57BL/6NCrl strain, the level of NKp46 was elevated following IL-12/IL-15/IL-18 incubation. In both mouse strains, NKG2D levels varied depending on the culture conditions, with a significant decrease following cGAMP and IL-12/IL-15/IL-18 incubation. Interestingly, regardless of mouse strain, CD69, an early activation marker, increased significantly when NK cells were cultured in the presence of IL-12/IL-15/IL-18 cytokines. NK cells isolated from C57BL/6NCrl mice showed a constant, approximately 50% level of NKG2A inhibitory receptor. We also assessed the intracellular level of granzyme B (GrB) (Fig. 2C). The highest production of GrB was observed following IL-15, IL-2/IL-15 and IL-15/cG activation in BALB/c mouse strain and IL-12/IL-15 (in C57BL/6NCrl). The addition of cGAMP to IL-12/IL-15/IL-18 cocktail did not influence the expression of activating receptors and GrB production (data not shown).

### NK cells intracellular cytokine profile

**Fig. 3:**
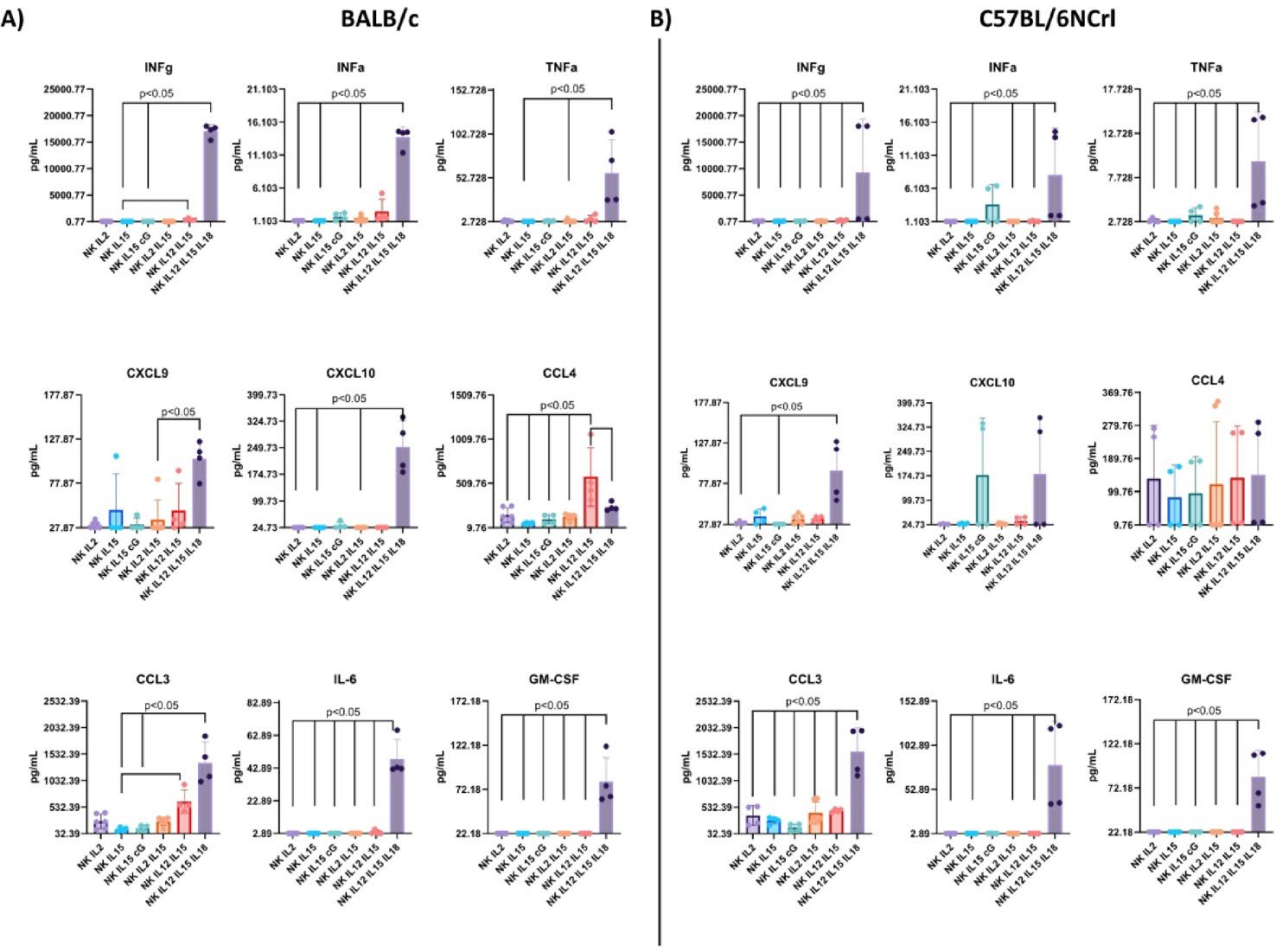
Intracellular cytokine profile of NK cells isolated from spleens of **(A)** BALB/c mice **(B)** C57BL/6NCrl mice. NK cells were lysed, and a bead-based immunoassay (Legendplex Mouse Cytokine Release Syndrome Panel) was performed to quantify 13 targets simultaneously. IL-10, IL-4, VEGF, and CCL2 levels were undetectable. The statistical significance: Kruskal-Wallis with Dunn’s multiple comparison test for non-parametric variables or one-way ANOVA with Tukey’s multiple comparisons test for parametric variables was performed, *p* < 0.05; *n* = 4.

Next, using multiplex flow cytometry-based immunoassay, we assessed the intracellular production profile of selected pro-inflammatory cytokines after 24-hour incubation with cGAMP and the tested cytokines (acc. to Fig. 3). NK cells were lysed, and samples were analyzed to determine the cytokine repertoire after specific activation. The most significant and exceptional change was the tremendous increase in production of INF-ɣ, INF-α, TNF-α, IL-6, GM-CSF cytokines, crucial for immune response regulation, and CXCL9, CXCL10, CCL4, CCL3 chemokines, key for directing the migration of immune cells, in both mouse strains but only following 24-hour IL-12/IL-15/IL-18 activation (Fig. 3A, B). IL-10, IL-4, VEGF, and CCL2 were undetectable. In particular, we observed an immense production of INF-ɣ (reaching almost 2 × 10^4^ pg/ml in BALB/c and 1 × 10^4^ pg/ml in C57BL/6NCrl). Other cytokines produced in large amounts after IL-12/IL-15/IL-18 activation were, in order of magnitude, CCL3 (up to 1500 pg/ml), CXCL10 (250 pg/ml in BALB/c; 170 pg/mL in C57BL/6NCrl), CXCL9 (app. 100 pg/ml), TNF-α (60 pg/ml in BALB/c; 10 pg/ml in C57BL/6NCrl), IL-6 (app. 50 pg/ml), GM-CSF (app. 70 pg/ml), INF-α (app. 10 pg/ml). CCL4 was produced abundantly after IL-12/IL-15 activation in BALB/c (over 500 pg/ml), whereas in C57BL/6NCrl, the level was similar in all conditions, reaching ~ 100 pg/ml. Due to cell death after incubation with cGAMP (Fig. 2A), the levels of cytokine production in this group were not shown in the graphs.

### Killing capacity of NK cells

**Fig. 4:**
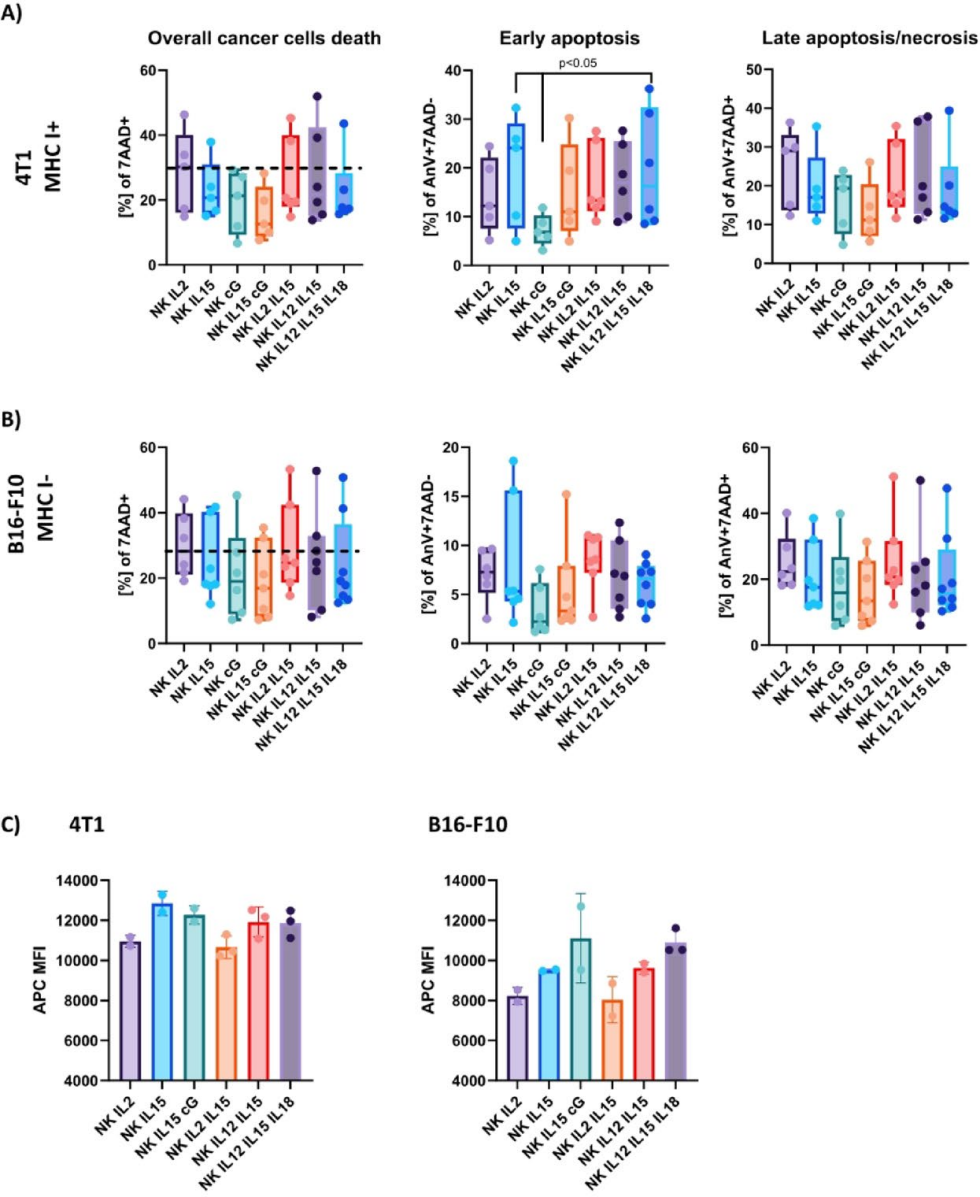
Killing capacity of NK cells isolated from spleens of BALB/c mice and C57BL/6NCrl mice towards **(A)** 4T1 breast carcinoma MHC class I^+^ and **(B)** B16-F10 melanoma MHC class I^−^. Death of cancer cells was assessed using AnV/7AAD test, *n* = 6–8. The statistical significance: Kruskal-Wallis with Dunn’s multiple comparison test for non-parametric variables, *p* < 0.05. **(C)** Proliferation rate was assessed with CellTrace Far Red and is expressed as mean fluorescence intensity (MFI). Slowly proliferating cells show a more intense APC signal.

To assess the ability of NK cells to kill cancer cells, magnetically separated NK cells were co-cultured with cancer cells. Freshly isolated NK cells of BALB/c strain were co-cultured with 4T1 breast carcinoma (MHC I^+^), and NK cells of C57BL/6NCrl strain with B16-F10 melanoma (MHC I^−^)^12^. Cancer cells were pre-stained with CellTrace Far Red Cell Proliferation Kit to evaluate the extent of proliferation inhibition. The tested activating cytokines were added to the co-cultures, and incubation was continued for 24 h. Cancer cells death and apoptosis were assessed using the Annexin V/7AAD test. We did not observe statistically significant differences in the ability of NK cells to eliminate cancer cells between groups activated with different cytokines, regardless of MHC I expression (Fig. 4A, B). However, a trend can be observed in the IL-2-treated group, in which the percentage of dead tumor cells increases for both MHC I^+^ and MHC I^−^ cells. In the IL-2-activated group, the percentage of late apoptotic/necrotic cancer cells (AnV^+^7AAD^+^) was also the highest. The rate of early apoptotic cancer cells (AnV^+^7AAD^−^) was higher in groups activated with IL-15 and IL-12/IL-15/L-18, but only in MHC I^+^ cells (Fig. 4A, B). The proliferation rate of 4T1 cancer cells incubated with NK cells was similar in all culture conditions (Fig. 4C). B16-F10 cells proliferated more slowly when incubated with NK cells in the presence of IL-12/IL-15/IL-18 and IL-15/cG activating cytokines (Fig. 4C). We did not observe cytotoxicity of the tested cytokines towards cancer cells (data not shown).

### Microscopic time-lapse observation of NK cells-induced cancer cells killing

**Fig. 5:**
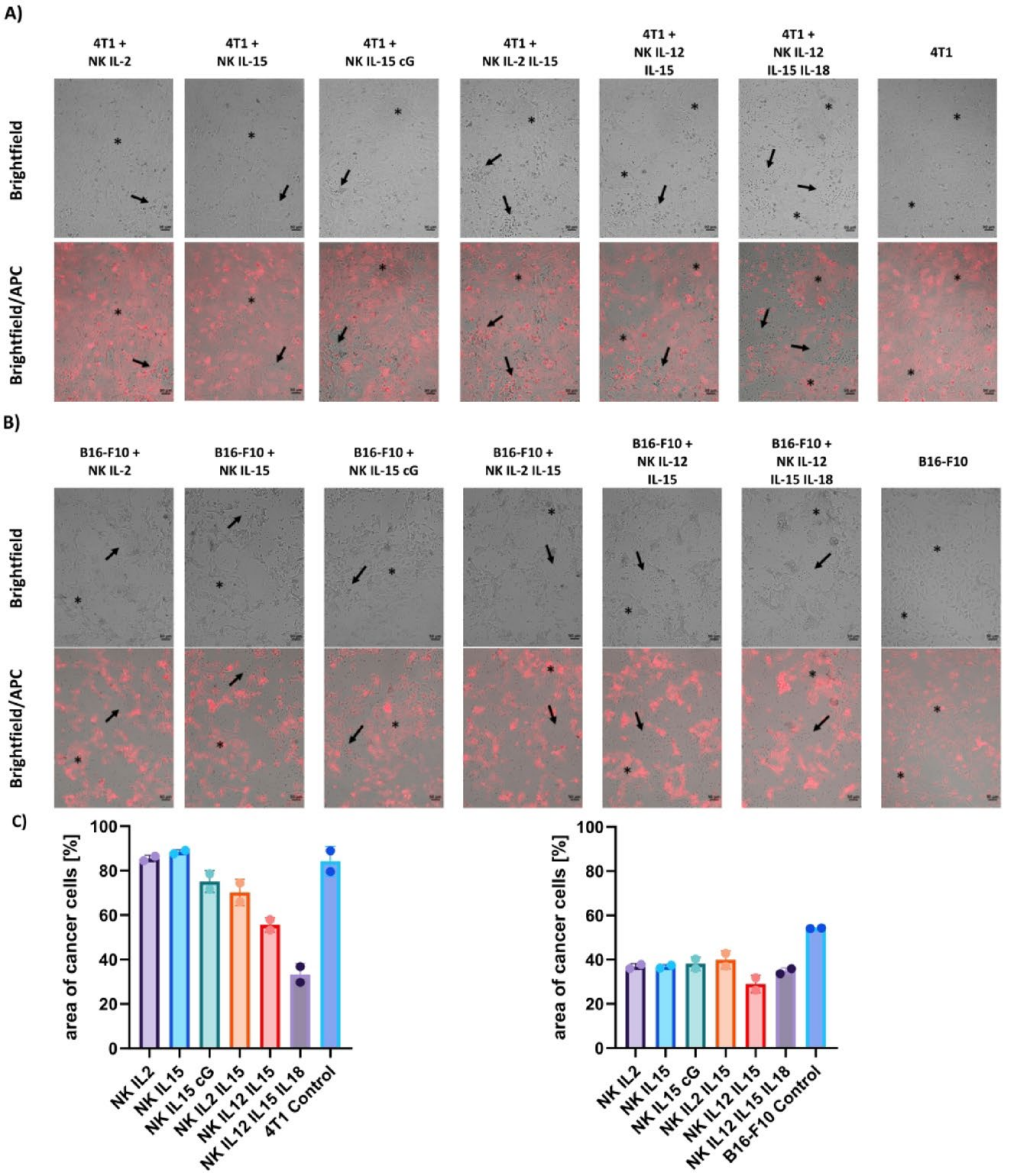
Representative microscopic images of NK cells co-cultures with **(A)** 4T1 breast carcinoma and **(B)** B16-F10 melanoma. Images were taken after 24 h of incubation. Upper panel: brightfield, lower panel: merged brightfield and APC channel. Images were taken using a 20× magnification, scale bar 50 μm.**(C)** The area covered by cancer cells was calculated using ImageJ.

Co-cultures of cancer cells with NK cells activated with tested cytokines were imaged on a time-lapse cell imaging microscope for 24 h. NK cells of BALB/c strain were co-cultured with 4T1 breast cancer cells (Fig. 5A), and NK cells of C57BL/6NCrl strain with B16-F10 melanoma (Fig. 5B). Cancer cells were pre-stained with CellTrace Far Red (APC channel). In the brightfield, NK cells appear as small, opalescent, high-contrast dots (arrow), while cancer cells appear as larger, flattened and elongated, with lower contrast (asterisk). In co-cultures with 4T1 breast carcinoma, we observed cytotoxic effect of NK cells activated with IL-2/IL-15, IL-12/IL-15, and IL-12/IL-15/IL-18 cytokines. In those conditions, the confluence of 4T1 cancer cells was reduced (Fig. 5C). However, only under conditions of NK cell activation with cytokines IL-12/IL-15 and IL-12/IL-15/IL-18 lysing effect was more visible, as evidenced by a lower density of tumor cells and a higher number of NK cells in the wells (Fig. 5A, C). In co-cultures with B16-F10 melanoma, all conditions of NK cell activation affected the morphology of cancer cells and reduced the density of tumor cells in the wells (Fig. 5B, C). The strongest cytotoxic effect, along with the highest number of NK cells, was observed in the groups after IL-12/IL-15 and IL-12/IL-15/IL-18 cytokine activation.

## Discussion

NK cell-based cancer immunotherapy is a promising field of research and development, encompassing strategies to enhance NK cells activity and persistence, as well as studies on combination therapies. Progress that has been made to enhance NK cells activity and function includes, among others, engineered CAR-NK, CRISPR-Cas9 edited NK cells, cytokines-enhanced NK cells, and long-lasting NK cells therapies based on memory-like phenotype^13,14^. Despite the great potential of NK cells in cancer immunotherapy, several limitations hinder their clinical use. One of the most important is that isolation techniques and expansion protocols show significant variation. Isolation and separation techniques are crucial for preserving the viability and purity of NK cells, while ex vivo expansion determines their immunophenotype and activation status required for their cytotoxic functionality^15^. Methods described in the literature typically employ the use of different cytokines, including interleukins IL-2, IL-12, IL-15, and IL-18, alone or in varying combinations.

Therefore, the aim of our study was to verify the viability of NK cells, their immunophenotype, cytokine production profile, and cytotoxic functionality after activation with the commonly used cytokines: IL-2, IL-15, IL-12, IL-18, or STING agonist cGAMP and their combinations. Direct comparison of NK cells isolated from the two mouse strains used as preclinical models of NK cells immunotherapy and their different activation conditions with commonly used cytokines is of great importance for the development of uniform standards for high-quality NK cell culture.

It has been shown that the addition of cGAMP enables STING activation and type I interferons (INFs-α, β) and INF-ɣ production in NK cells, thereby supporting the activation of NK cells in vitro^16^. In our previous studies, we demonstrated a significant increase in intratumoral NK cells infiltration after administration of the STING agonist - cGAMP, but not necessarily in cytotoxic T cells^12,17^. Moreover, cGAMP enhanced the sensitivity of pancreatic cancer cells to NK cell cytotoxicity and, when combined with CAR-NK-92 cells targeting mesothelin, improved anti-tumor efficacy^18^. In our study, we have observed dramatically reduced viability of NK cells after 24 h incubation with cGAMP, with reduced expression of activating receptors and granzyme B production. Consequently, we did not observe an increase in INFs production. Therefore, prolonged cGAMP incubation deteriorated NK cells function. Another study indicated that indirect treatment of NK cells with an encapsulated form of the STING agonist did not result in NK cell death but activated them more effectively than soluble cGAMP^19^. In our study, the addition of IL-15 rescued, to some extent, NK cells from cGAMP-induced cell death and resulted in increased levels of activating receptors and executive molecule granzyme B. The appropriate, optimized dose and time of NK cells activation with cGAMP is crucial to obtain cells with potentially clinically relevant quality. The use of cGAMP as a compound activating the immune system in anticancer therapy should assume the parallel use of cytokines protecting NK (e.g. IL-15 or IL-12/IL-15/IL-18 cytokines cocktail) cells against the toxic effects of cGAMP.

IL-2 and IL-15 are the two cytokines most commonly used for ex vivo NK cells expansion protocols^20–23^. Both cytokines are known to induce ex vivo NK cell proliferation and activation with an increase in the expression of activating receptors and also an improvement of their cytotoxic functions^24^. In our study, we have observed higher viability of cells incubated for 24 h with IL-15 compared to IL-2. The expression of activating receptors and granzyme B was also slightly higher in IL-15-treated NK cells. The combination of both cytokines provided similar effects. Both IL-2 and IL-15, or their combination, failed to induce increased production of pro-inflammatory cytokines. Only the addition of IL-12 and IL-18 to IL-15 augmented the production of INF-ɣ and the other cytokines able to modulate functions of innate and adaptive immunity.

We observed a massive production of pro-inflammatory cytokines, especially INF-ɣ, when NK cells were cultured with IL-12, IL-15, and IL-18. It has been shown that stimulation of immature NK cells with a cytokine cocktail containing IL-12, IL-18, and IL-15 leads to differentiation into memory-like NK cells, termed cytokine-induced memory-like (CIML) NK cells^25–27^. CIML NK cells, in contrast to short-lived NK cells, exhibit a long-term life span with adaptive immunity characteristics^28^. CIML NK cells are characterized by enhanced INF-γ production, NK cells persistence in vivo, target recognition, and effector functions^29,30^. First-in-human phase 1 clinical trial showed that adoptively transferred CIML NK cells proliferated and expanded in acute myeloid leukemia patients and demonstrated robust responses against leukemia targets^30^. Our study confirmed a vast production of INF-ɣ along with other pro-inflammatory cytokines and chemokines. The viability of CIML NK cells was moderate (50–60% of live cells). We have also observed a significant increase in CD69 expression in CIML NK cells. It is in line with observations that CD69, an early activation marker, influences NK cells function, including cytotoxicity and cytokine production^31,32^. In our study, however, similarly to the study by Cooper et al^25^., we observed that CIML NK cells produced low levels of granzyme B protein and did not kill target cells more effectively than differentially activated NK cells. Further studies on the clinical application of CIML NK cells should be conducted, assessing their therapeutic potential in human cancer models. Performing such studies on human NK and cancer cells is a necessary step to translate the results into the clinic.

We also observed a major production of CCL3 and CXCL9, CXCL10 chemokines by CIML NK cells. It has been shown that CCL3 and CCL4 are potent chemoattractants for immature DCs, inducing dendritic cells (DCs) migration to the tumor site and their maturation^33^. Moreover, NK cells are a major lymphocyte subtype that is preferentially recruited to the CCL3-rich tumor^34^. CCL3-recruited NK cells can further produce in tumors INF-γ and TNF-α, which synergistically augment the production of CXCL9 and CXCL10 chemokines^35^. In turn, CXCL9, CXCL10 chemokines enhanced T cell recruitment, guiding the infiltration of CXCR3^+^ NK and T cells into solid tumors. Even though we did not observe a significant increase in NK-cell-mediated cancer cells killing in vitro, we conclude that ex vivo activated CIML NK cells, due to the secretion of an abundant amount of cytokines, may amplify innate and adaptive anti-tumor immunity. It has been shown that enhanced NK and T cells infiltration is associated with favorable responses to chemotherapy and immunotherapy with immune checkpoint blockers^36^. Additionally, *ex vivo-activated* NK cells did not secrete immunosuppressive cytokines such as IL-10, which suggests that external activation of NK cells can be an effective anti-cancer therapy.

The activity of NK cells is based on the recognition of MHC class I molecules. Healthy cells typically express normal levels of MHC class I molecules, which are recognized by NK inhibitory receptors such as NKG2A. Many cancer cells downregulate MHC expression, escaping from cytotoxic CD8^+^ T cells. On the other hand, the reduced or absent expression of MHC I, typical for malignant cells, makes them potential targets for NK cell-mediated cytotoxicity^15^. This suggests that NK cells and CD8^+^ T cells compensate for each other against tumor cells^37^. The activation of NK cells is controlled by a balance between signals from inhibitory and activating receptors. MHC I-expressing tumor cells can also be killed by NK cells, through their induced expression of certain activating receptors, including NKG2D, NKp46, and NKp44^37^. In our study, we used two MHC I^+^ and MHC I^−^ tumor cell lines to verify the cytotoxic functions of NK cells after their activation with various cytokines and their combinations. Unfortunately, we failed to show a significant difference in NK cells specific cancer cell killing. We observed almost a constant specific cell death at the level of 30% regardless of MHC class I status. Although the lack of significant increase in NK cells-induced cancer cells apoptosis and necrosis following cytokine cocktail incubation, our microscopic observations suggest increased cancer cells lysis. Importantly, in the case of B16-F10 MHC I- cells, the destruction of cancer cells was observed regardless of the type of cytokines used, proving high NK cells killing potential toward cells with downregulated MHC I, which are unable to bind inhibitory receptors of NK cells.

The use of appropriate inducers of isolated NK cells may be an effective method for their activation. The most promising combination is that of IL-12, IL-15, and IL-18 cytokines. This combination activates NK cells and increases the secretion of pro-inflammatory cytokines. Activation of NK cells, particularly using a combination of IL-12, IL-15, and IL-18 cytokines, inhibited the growth of both MHC^+^ and MHC^−^ tumor cells and caused their lysis.

## Materials and methods

### Mice and ethics statement

In parallel studies, organs were collected for immunohistochemical analysis, and spleens were donated for NK cell isolation. For this purpose, the mice were euthanized by cervical dislocation and the organs necessary for analysis were collected, including the spleens. Experiments were conducted on female BALB/c and C57Bl/6NCrl mice (8–10 weeks old) obtained from a breeding facility at the National Research Institute of Oncology, Gliwice branch (Gliwice, Poland). Experiments on animals were carried out with the consent of the Local Ethical Committee for Experiments on Animals at the Medical University of Silesia in Katowice (permissions No: 6/2024). Mice were housed in the Maria Sklodowska-Curie National Research Institute of Oncology, Gliwice Branch (Poland) in a pathogen-free facility in SPF standard in a HEPA-filtered Allentown’s IVC System (Allentown Caging Equipment Co, NJ, USA). The animals received a total pathogen-free standard diet (Altromin 1314, Altromin Spezialfutter GmbH and Co. KG, Lage, Germany) and water ad libitum throughout the whole study. Animals were treated in accordance with the recommendations in the Guide for the Care and Use of Laboratory Animals of the National Institutes of Health. Experiments on animals were conducted in accordance with the 3R rule. The study adhered to the ARRIVE guideline.

### NK cells isolation and magnetic sorting

Spleens were mashed using an ACK solution moisturized cell strainer (70-µm nylon mesh), and a syringe plunger. The residues of spleens left on the strainer were rinsed with 2 ml ACK solution (Thermo Fisher Scientific). The obtained splenocytes were washed with pre-cooled PBS supplemented with 2% FBS, centrifuged at 300 × g for 10 min, and passed through a 40-µm strainer. Splenocytes were processed for magnetic sorting with the use of the NK cell isolation kit (cat. No. 130-115−818, Miltenyi Biotec, Bergisch Gladbach, Germany) according to the manufacturer’s instructions. The QuadroMACS Separator and the LS columns were used (Miltenyi Biotec). Briefly, splenocytes (1 × 10^8^ cells/ml) were suspended in MACS buffer containing PBS, 0.5% FBS, and 2mM EDTA and incubated with NK cell biotin-antibody cocktail and anti-biotin microbeads, respectively. The purity of the obtained NK cells was verified by flow cytometry immediately after each NK cells sorting procedure.

### NK cells culture and flow cytometry phenotype assessment

Immediately after NK cells isolation, the cells were plated in a 96-well plate in complete RPMI medium containing 10% heat-inactivated FBS and 1% penicillin-streptomycin at a density of 3 × 10^5^ for 24 h. NK cells were cultured in the presence of IL-2, IL-12, IL-15, IL-18 cytokines at a concentration of 1000 U/ml, 10 ng/ml, 10 ng/ml, 100 ng/ml, respectively, or cGAMP (10 µM; cG) according to allocated groups. 24 h after plating, the phenotype of NK cells was assessed. The isolated cells were blocked with anti-mouse CD16/32 (clone: 93) antibody (BioLegend) and stained with the following extracellular antibodies: anti-CD45 (clone: 30-F11), anti-CD3 (clone:17A2), anti-CD49b (clone: DX5), anti-NKp46 (clone: 29A1.4), anti-CD69 (clone: H1.2F3), anti-NKG2D (clone: CX5), anti-GrB (clone: QA18A28) (BioLegend); anti-NKG2A (clone: REA1161) (Miltenyi Biotec). Intracellular staining of granzyme B was performed using the Fix/Perm Kit (eBioscience) using the manufacturer’s instructions. Dead cells were stained using DAPI (Merck). In flow cytometry analyses (BD FACSCanto II, Becton Dickinson, Franklin Lakes, NJ, USA), gates dividing negative from positive cells were based on fluorescence minus one (FMO) controls.

### NK cells intracellular cytokines assessment

Following 24-hour incubation with cytokines, cytokine-activated NK cells were centrifuged at 300×g, 10 min, 4 °C. The cells were stored at − 80 °C before lysing. The cells were lysed with the IP buffer (Thermo Fisher Scientific) supplemented with protease inhibitors containing EDTA (Merck). The lysates were collected and stored at − 80 °C until further analysis. The type and quantity of cytokines have been assessed with LEGENDplex™ Mouse Cytokine Release Syndrome Panel (Biolegend) by BD FACSCanto II flow cytometer (Becton Dickinson, Franklin Lakes, NJ, USA), according to the manufacturer’s instructions. The data were analysed using LEGENDplex™ Data Analysis Software Version 8.0 (BioLegend).

### NK cells in vitro cytotoxicity assessment

4T1 murine breast carcinoma and B16-F10 murine melanoma were pre-stained with CellTrace Far Red Cell Proliferation Kit (Thermo Fisher Scientific) according to the manufacturer’s instructions. Briefly, cancer cells were stained with 1 µM CellTrace Far Red staining solution and incubated for 15 min in a 37 °C. The unbound dye was washed out using complete medium. Cancer cells were plated at a density of 3 × 10^4^ per well of a 96-well plate for 4 h in complete RPMI medium containing IL-2, IL-12, IL-15, and IL-18 cytokines at a concentration of 1000 U/ml, 10 ng/mL, 10 ng/ml, 100 ng/ml, respectively, or cGAMP (10µM; cG) according to allocated groups. Following 4 h (time of cancer cells attachment to the plate surface), freshly isolated NK cells were added to individual wells at a density of 7.5 × 10^4^ (2.5:1 effector to target cell ratio). Co-cultures were imaged on time-lapse microscopy with a controlled environmental chamber for 24 h. Following 24 h, floating NK cells and cancer cells were collected using 0.25% trypsin. Collected co-cultures were stained with 5 µl Annexin V and 5 µl 7AAD (Biolegend) to assess cell viability by flow cytometry.

### Statistical analysis

The distribution’s normality was verified using the Shapiro–Wilk test. The homogeneity of variance was checked using the Brown–Forsythe and/or Levene’s tests. If assumptions for parametric testing were met, a one-way ANOVA was performed, followed by post-hoc comparisons using Tukey’s HSD or LSD tests. For variables not meeting the conditions of parametric testing, the Kruskal-Wallis test with Dunn’s multiple comparisons test was applied. Statistical significance was marked with asterisks. p-values < 0.05 were considered statistically significant. The data are shown as mean ± SEM (Standard Error of Mean). All analyses were conducted using GraphPad Prism V.10.5.0 software (GraphPad Software, Inc., La Jolla, California, USA).

## Data Availability

All data relevant to the study are included in the article. Data are available upon reasonable request from the corresponding author.
